# 

**Published:** 1881-03

**Authors:** 


### TABLE OF CONTENTS,

### ORIGINAL COMMUNICATIONS.

Malarial Poison. By W. O'Daniel, A.M., M.D., of Bullard's, Ga..... 713
Valedictory Address of M. H. O'Daniel, of Bullard's, Ga................ 716
A Case of Occulsion of the Vagina. By Jno. G. Earnest, M.D., of Newnan, Ga.................................................................. 722
Inaugural Thesis on the Action of Remedies. By Robert O Cotter 725

### REPORTS OF SOCIETIES.

Cincinnati Academy of Medicine.............................................. 740
Medical Society of the County of New York........................................ 744

### SELECTIONS.

The Hystero Neurosis of the Stomach in Pregnancy. By John S. Warren M D., of New York.............................................. 751
Parallelism of the Eruptive Fevers. By M Jules Simon................ 758
Dipsomania. By M. J. Halloran, M.D., Worcester, Mass................ 761

### EDITORIAL.

The Medical Profession and Advertising.................................... 765

### BIBLIOGRAPHICAL

A Practical Treatise on Diseases of the Skin................................ 766
A Manual of Diseases of the Throat and Nose ................................ 766
Syphilis and Marriage.............................................................. 767
A Treatise on the Materia Medica and Thereapeutics of the Skin.... 767
A Treatise on Albuminuria......................................................... 767
Pamphlets Received.................................................................. 768

### EXCERPTA.

The Bloodless Method of Performing Surgical Operations.............. 769
Antiseptic Dressings.................................................................. 770
Pilocarpine in Cutaneous Diseases............................................. 771
Pruritus Vulvæ Relieved by Iodide of Potassium ......................... 772
New Procedure in the Operation for Phimosis.............................. 772
Restoration of an Asphyxiated Infant.......................................... 773
Prolonged Gestation.................................................................. 773
Index to Volume XVIII................................................................ 774

